# Intestinal microbiome is related to lifetime antibiotic use in Finnish pre-school children

**DOI:** 10.1038/ncomms10410

**Published:** 2016-01-26

**Authors:** Katri Korpela, Anne Salonen, Lauri J. Virta, Riina A. Kekkonen, Kristoffer Forslund, Peer Bork, Willem M. de Vos

**Affiliations:** 1 Immunobiology Research Program, Department of Bacteriology and Immunology, University of Helsinki, Haartmaninkatu 3, PO Box 21, 00014 Helsinki, Finland; 2 Research Department, Social Insurance Institution, Turku, Peltolantie 3, 20720, Finland; 3 Valio Limited, R&D, Meijeritie 4, 00370 Helsinki, Finland; 4 European Molecular Biology Laboratory, PO Box 1022.40, 69012 Heidelberg, Germany; 5 Department of Veterinary Biosciences, University of Helsinki, PO Box 66, 00014 Helsinki, Finland; 6 Laboratory of Microbiology, Wageningen University, Dreijenplein 10, 6703 HB Wageningen, The Netherlands

## Abstract

Early-life antibiotic use is associated with increased risk for metabolic and immunological diseases, and mouse studies indicate a causal role of the disrupted microbiome. However, little is known about the impacts of antibiotics on the developing microbiome of children. Here we use phylogenetics, metagenomics and individual antibiotic purchase records to show that macrolide use in 2–7 year-old Finnish children (*N*=142; sampled at two time points) is associated with a long-lasting shift in microbiota composition and metabolism. The shift includes depletion of Actinobacteria, increase in Bacteroidetes and Proteobacteria, decrease in bile-salt hydrolase and increase in macrolide resistance. Furthermore, macrolide use in early life is associated with increased risk of asthma and predisposes to antibiotic-associated weight gain. Overweight and asthmatic children have distinct microbiota compositions. Penicillins leave a weaker mark on the microbiota than macrolides. Our results support the idea that, without compromising clinical practice, the impact on the intestinal microbiota should be considered when prescribing antibiotics.

## Abstract (web summary)

The impact of antibiotics on the microbiome and health of children is poorly understood. Here, Korpela *et al*. study the gut microbiome of 142 children and show that the use of macrolides, but not penicillins, is associated with long-lasting shifts in microbiota composition and increased risk of asthma and overweight.

## Graphical abstract

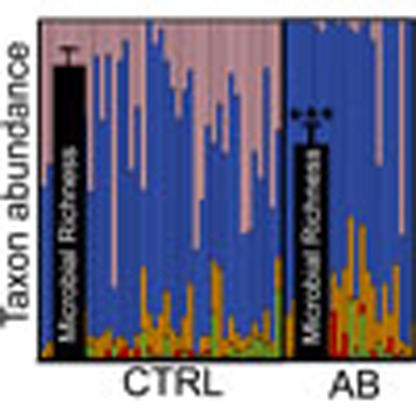

Antibiotics are the most commonly used drugs in the pediatric populations of western countries^1^,^2^. Early-life antibiotic use is associated with increased risk for inflammatory bowel disease^3^, overweight^4^,^5^,^6^ and asthma^7^. Experimental mouse studies have linked early-life administration of antibiotics with metabolic and immune-related diseases, possibly via changes in the intestinal microbiome^8^,^9^,^10^,^11^. However, studies with mice are hard to extrapolate to human because of marked differences in metabolism, diet and microbiome^12^. In trials with human adults, oral intake of antibiotics has been shown to markedly affect the intestinal microbiota, reducing the diversity and altering the composition^13^,^14^. However, these studies are limited by small sample size (3–4 individuals) and are complicated by large inter-individual variation in microbiota composition. In neonates, the early development of the microbiota differs between antibiotic-treated and non-treated infants^15^,^16^, but nothing is currently known about the long-term associations between lifetime antibiotic use and the intestinal microbiome in children. Furthermore, antibiotic resistance is of major public health concern globally and is expected to become an increasingly serious obstacle in the treatment of infections^17^. To better understand the health implications of early-life antibiotic use, it is important to consider how different types of antibiotics influence the intestinal microbiome. In Finland, the Social Insurance Institute maintains a national database on prescription drug purchases and eligibility for special reimbursement due to chronic diseases linked with personal identification information of the patient^18^. Here we utilize this unique database and pyrosequencing of fecal microbiomes to investigate the short- and long-term effects of antibiotics on pre-school children's intestinal microbiome and health.

We show that antibiotic use in childhood is associated with marked changes in the intestinal microbiota composition, which persist for over 6 months. Macrolides, particularly, appear to modify the microbiota and their functions, being the strongest driver of inter-individual differences in microbiota composition in our cohort. Among the children who received macrolides in early life, we find a positive correlation between overall lifetime antibiotic use and body mass index (BMI), as well as an increased risk of asthma, suggesting that macrolide use may alter the microbiota in infants in a way that predisposes to antibiotic-associated weight gain and asthma in later childhood.

## Results

### Microbiota composition correlates with macrolide use

Our cohort consists of 236 Finnish children aged 2–7 years (median age 5 years), attending day care at the time of the study. A total of 257 fecal samples were obtained from 142 children (Supplementary Fig. 1) who donated either a single sample (27 children) or two samples (115 children; samples were taken 7 months apart). The fecal samples were grouped based on antibiotic use (Supplementary Table 1). As the control group, we selected samples from children who had not used antibiotics for >2 years and whose total lifetime antibiotic use was <1 course per year on average. The microbiota composition of the other groups was compared with that of the control group.

**Figure 1:**
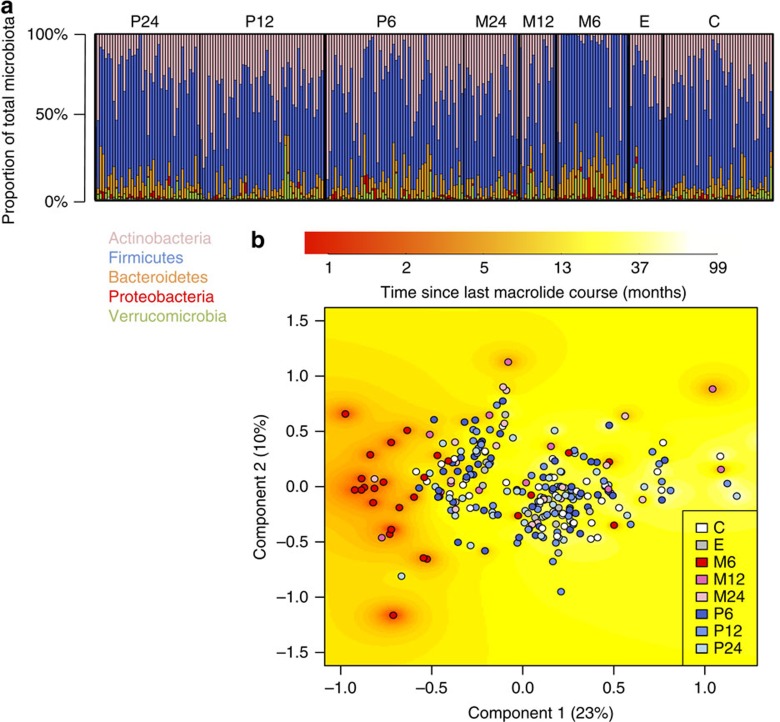
Microbiota composition in 257 fecal samples as arranged per group. C denotes control group, no antibiotics for the past 2 years and in total <1 course per year of life on average. E denotes early-life exposure group, no antibiotics for the past 2 years and >1 course per year of life on average. M6 denotes macrolide course within 6 months; M12 denotes macrolide course within 6–12 months; M24 denotes macrolide course within 12–24 months. P6, P12 and P24 denote penicillin courses within 6, 6–12 and 12–24 months, respectively. (**a**) Phyla composition. (**b**) Genus-level microbiota composition according to PCoA analysis. The background colour indicates interpolated time since the last macrolide course.

Although the indications for penicillin and macrolide courses were the same—both were prescribed mainly for respiratory infections—macrolide use specifically was associated with a clear shift in the microbiota composition (Fig. 1). The group M6 (exposed to macrolides within 6 months) had a distinct microbiota composition evident even at the phylum level (Fig. 1a). The abundance of Actinobacteria, which includes *Bifidobacterium* as the dominant genus, was reduced in this group, in many cases to below detectability, and the abundances of the Gram-negative phyla Bacteroidetes and Proteobacteria were increased. These changes were largely resolved in the M12 and M24 groups, indicating that the phylum-level balance of the microbiota recovered within 1 year after a macrolide course. The penicillin-user groups (P6, P12 and P24) did not have a distinctly different phyla composition. The genus-level principal coordinates analysis (PCoA) indicated that the main driver determining the overall microbiota composition was macrolide use: most of the M6 samples clustered together with low scores on component 1, whereas the rest of the samples showed less clear patterns (Fig. 1b). The time since the last macrolide course correlated with component 1 (*r*=0.36, *P*<0.001), indicating that the beta diversity among these samples was associated with recovery from the most recent macrolide course. Penicillin use was not strongly associated with the principal components. The same patterns were evident in both the 2–4- and 5–7-year-old children, indicating that the dominant effect of macrolide exposure on the microbiota was not age-dependent. Furthermore, the pattern was evident regardless of previous antibiotic exposure background. We thus conclude that macrolides appear to set the microbiota to a certain state, regardless of the baseline composition.

### Long-lasting microbiome shifts after antibiotic use

**Figure 2:**
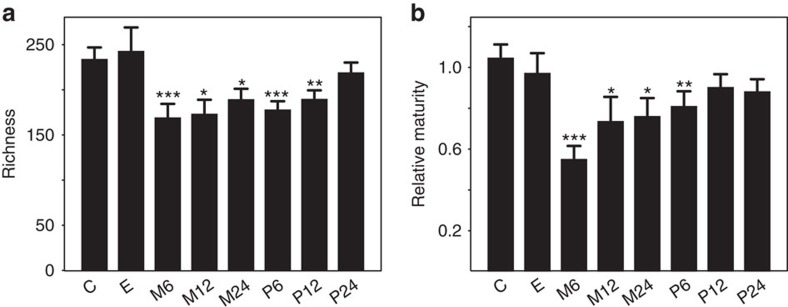
Microbiota richness and maturity relative to age in the different groups. (**a**) Number of species-like phylotypes. (**b**) Score based on 24 age-associated genera (Supplementary Table 2). Asterisks indicate significance of the difference to the control group ‘C'. *P<0.05, **P<0.01 and ***P<0.001, estimated using linear models. Mean values are shown and the error bars show the s.e. values. The number of samples in both panels is 257 (Supplementary Table 1).

Antibiotic use, and macrolide use in particular, was associated with a long-term reduction in microbial richness, which did not reach the level of the control samples even 12–24 months after the course (Fig. 2a). The age-adjusted maturity index (Supplementary Table 2) was also reduced in the samples exposed to macrolides within 2 years, and in group P6, indicating that the age-associated aspects of the microbiota recovered within 6–12 months after a penicillin course, but did not fully recover from a macrolide course even after 2 years (Fig. 2b).

After controlling for age, BMI *z*-score and health (asthma, allergic dermatitis or parent-reported allergies), 18 of the 48 genera and 7 of the 14 orders that were detected in >30% of the samples were significantly associated with recent antibiotic use, that is, were significantly different in the groups M6 or P6 as compared with the control group (Supplementary Table 3 and Supplementary Fig. 2). Four of the six commonly detected genera belonging to Actinobacteria were associated with macrolide use: one increased 10-fold (*Eggerthella*) and three decreased 4–10-fold (*Bifidobacterium*, *Collinsella* and unassigned *Coriobacteriaceae*) in the group M6. Of these, *Collinsella* was significantly reduced in all groups compared with the control group, suggesting that this genus is strongly affected by antibiotics. Among the Bacteroidetes, *Bacteroides* was twofold elevated in the group M6 and *Parabacteroides* in the groups M6, M12, P6 and P24. Among the other Gram-negative bacteria, a twofold increase in Proteobacteria in the group M6 was notable, but apparently distributed among several genera with a member of *Enterobacteriaceae* as the most significantly affected (*P*=0.05 in a generalized linear model). Among the Firmicutes, bacteria from the order Gemellales were significantly and consistently reduced in the penicillin-user groups. The order Lactobacillales was increased in all macrolide user groups. This was largely owing to an increase in *Streptococcus*, whereas *Lactobacillus* was decreased. *Lactobacillus* was also decreased in the groups P6 and P12. Erysipelotrichales as an order, and its member genus *Eubacterium*, were elevated in the group M6. An unassigned member of *Clostridiales*, a member related to *Christensenella*, and *Anaerostipes* were reduced in the group M6, whereas the genera *Clostridium* and *Dorea* were increased.

In addition to stratifying the children by the most recent antibiotic course, we investigated the possibility of cumulative effects of lifetime antibiotic exposure (number of antibiotic courses per year of life). Recent antibiotic use correlated with lifetime use: all antibiotic user groups had higher lifetime use than the control group, as the control group was selected partly based on low lifetime antibiotic use (*P*<0.001 in a generalized linear model). Hence, after controlling for recent antibiotic use, the cumulative lifetime use had a minimal association with the microbiota composition. Four genera, all in the order Clostridiales, were significantly positively associated with total lifetime macrolide use (*Blautia, P*=0.002; *Dorea, P*=0.002; *Dialister, P*=0.004; and *Megamonas, P*=0.006). *Megamonas* was also positively associated with lifetime penicillin use (*P*=0.003). Microbial richness was not associated with lifetime antibiotic use after controlling for the most recent antibiotic course.

**Figure 3:**
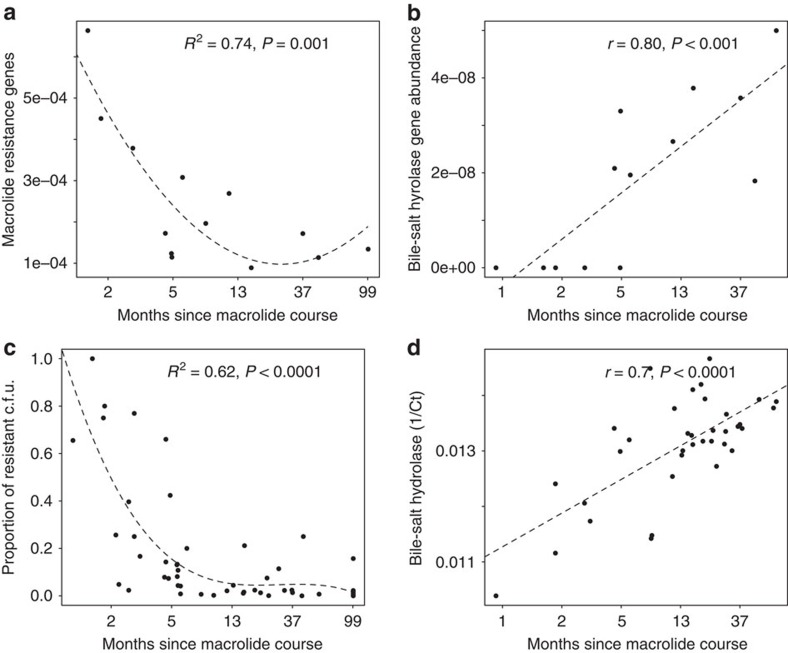
Macrolide resistance and bile-salt hydrolase abundance in relation to time since the last macrolide course. The dashed lines show the model fit (linear or polynomial), *R*^2^ indicates the variation explained by the model and the *P* values (estimated using linear models) are indicated. (**a**) Macrolide resistance potential inferred from metagenomic analysis, *N*=14. (**b**) Bile-salt hydrolase abundance in metagenomes, *N*=14. (**c**) Macrolide resistance measured as proportion of anaerobic c.f.u.'s growing with erythromycin compared with c.f.u. without erythromycin, *N*=80. (**d**) Combined relative abundance of three bile-salt hydrolase genes (*bsh*) based on qPCR as a function of time since last macrolide course, *N*=37.

Based on the metagenomic analysis, we discovered that macrolide resistance was high in the recently macrolide-exposed microbiomes, and bile-salt hydrolases were reduced (Fig. 3a,b). The culture-based macrolide resistance testing revealed the same pattern: macrolide resistance was initially elevated after a macrolide course but declined linearly until it reached a low baseline level at ca. 6–12 months after the course (Fig. 3c). Moreover, a quantitative PCR (qPCR)-based assay on 130 fecal samples showed that the abundance of *erm*F and *erm*B also correlated negatively with time since the last macrolide course (*r*=−0.31, *P*=0.005 in Pearson correlation test). There was a clear positive correlation between qPCR-measured abundance of *bsh* genes and time since the last macrolide course (Fig. 3d).

### Macrolide use associated with asthma and overweight

Early-life antibiotic use was associated with health outcomes. Current or developing asthma was significantly positively associated with frequent macrolide use during the first 2 years of life: odds ratio for the group that received >2 macrolide courses (*N*=32) compared with the non-exposed (*N*=116) was 6.11 (95% confidence interval: 1.53–26.58, *P*=0.004 in Fisher's test). We also observed a strong correlation between total lifetime antibiotic use and the BMI *z*-score in the children with >2 macrolide courses before the age of 2 years (Supplementary Fig. 3), but not in the non-exposed children.

We compared the microbiota compositions of asthmatic (*N*=8) and overweight (*N*=9) children with those of matched healthy and normal-weight controls. The asthmatic cases were distinguished from the healthy controls by a set of three bacterial genera with significantly different abundances: *Blautia, Rothia* and *Coprobacillus* (Supplementary Fig. 4A). The overweight cases were distinguished from the matched normal-weight controls by a set of four bacterial groups: *Clostridium (Erysipelotrichaceae), Clostridium (Clostridiaceae), Akkermansia* and *Enterococcus* (Supplementary Fig. 4B).

## Discussion

Our results show that macrolide use in childhood is associated with long-term distortions in the composition, function and antibiotic resistance of the intestinal microbiota. The use of penicillin-type antibiotics was not associated with large microbiome-wide functional or compositional changes in the microbiota.

We observed clear differences in the microbiota composition in the antibiotic-treated children as compared with those who were not exposed to antibiotics for >2 years. Some aspects of the microbiota, such as the abundance of *Bifidobacterium* and *Bacteroides*, and macrolide resistance, normalized within 12 months after a macrolide course. However, the abundances of *Collinsella, Lactobacillus* and *Anaerostipes*, as well as the total richness and maturity of the microbiota, remained reduced for up to 2 years after a macrolide course. A full recovery of the microbiota from an antibiotic course thus appeared to take longer than the average interval between courses (the children received on average 1.8±1.5 courses per year). When antibiotics are used annually or more frequently, the microbiota may not have time to recover and the antibiotic-associated microbiota composition may persist.

The children receiving antibiotics had infections, whereas those not receiving antibiotics did not have bacterial infections, requiring antibiotic treatment—although they may have had viral infections— and hence we considered the possibility that the microbiota changes could be attributed to the infections. However, both macrolide and penicillin antibiotics were prescribed mostly for respiratory infections, but were associated with clearly different, even opposite, changes in the microbiota. Furthermore, it has previously been shown that fever does not alter the microbiota composition of children^19^. This indicates that infections alone do not explain all of the observed changes. Finally, the strong correlation between macrolide use and macrolide resistance suggests a causal relation with the used antibiotics. Experimental studies with animals^8^,^9^,^20^ and with adult humans^13^,^14^ have shown that the intake of antibiotics alters the intestinal microbiota. In a recent experiment with mice^11^, early-life macrolide and penicillin treatment was shown to cause very similar effects on the microbiota as we observed, strongly suggesting causality in our cohort as well.

In an earlier mouse study, it was shown that although the microbiota recover when antibiotic administration is ceased, the metabolic changes persist^10^. Our results confirm corresponding patterns in human children: the children with heavy early-life use of antibiotics but no antibiotics for at least 2 years before sample donation had a microbiota similar to those with low lifetime antibiotic use. Nevertheless, early-life use of macrolides predisposed to overweight and asthma. These results suggest that even transient microbiome disturbance in early life may have long-term effects on the metabolic and immunological health of the child.

We found a strong positive association between recorded antibiotic use and BMI *z*-scores, specifically in a group of children that were exposed to macrolides in early life. The growth-promoting effect of antibiotics in animals^21^ and in children of low-income countries^22^,^23^ have been linked with a reduction in subclinical infections, although a role for the intestinal microbiota was proposed already in the 1950s (ref. 22). In western children^4^,^24^, a reduction in subclinical infections is unlikely to explain the association between antibiotic use and BMI *z*-scores. Furthermore, it does not explain why priming specifically with macrolides predisposes to the antibiotic-associated weight gain. Rather, the weight gain is likely to occur via macrolide-associated changes in the microbiota, which promote excessive weight gain, as demonstrated experimentally with mice^11^. Early-life macrolide treatment in mice lead to reduced level of ghrelin, changes in hepatic gene expression and an inability of the microbiome to adapt to dietary changes^11^. For the first time in human children, we demonstrate antibiotic-associated changes in the microbiome, which have previously been associated with metabolic diseases and obesity: reduced diversity^25^, increased abundance of endotoxin-producing (Gram-negative) organisms, such as Proteobacteria^26^, as well as depletion of *Bifidobacterium*^27^, *Christensenella*^28^ and bile-salt hydrolase producing organisms^29^.

Bile acid metabolism is one of the key functions performed by the intestinal bacteria, with strong effects on host energy metabolism^30^. Bacterial bile-salt hydrolases de-conjugate primary conjugated bile acids, rendering them susceptible for further bacterial modification into secondary and tertiary forms^31^. Bile-salt hydrolases are enriched in the human intestinal microbiome^32^, indicating their importance in the human digestive system. Modified bile acids function as metabolic regulators, and bile-salt hydrolase activity of the microbiota has been shown to reduce host weigh gain, insulin resistance and blood cholesterol via FXR-α and TGR5 signalling^29^,^33^.

Previous birth-cohort studies and large international studies have found an association between asthma and early-life antibiotic use^34^, particularly with respect to cephalosporins^35^ and macrolides^36^. Increased risk of allergic disease has been associated with deviations in the microbiota characteristics in early life, such as depletion of *Lactobacillus* and/or *Bifidobacterium*^37^,^38^,^39^, colonization with *Bacteroides fragilis*^40^ and members of Proteobacteria^41^, as well as low diversity^42^. All of these characteristics were present in the recently macrolide-treated children in our cohort, suggesting that macrolide use alters the microbiota in a way that disrupts the healthy immune system development. Furthermore, other factors, which alter the microbiota in a similar manner, such as Caesarean section, lack of breastfeeding and pre- and perinatal stress, predispose to asthma^43^. A specific case is the 10-fold increased levels of *Eggerthella* spp. observed after macrolide exposure. Most *Eggerthella* spp. are pathogens^44^ and may promote an inflammatory response. Experimental evidence from animal models shows that antibiotics in early life disrupt the microbiota and thereby the development of the immune system, leading to airway hyper-responsiveness in susceptible individuals^9^,^45^.

To conclude, macrolide use was associated with microbiota characteristics that have previously been associated with the risk of immunological and metabolic diseases, as well as obesity. Furthermore, macrolide use promoted a marked increase in macrolide resistance of the microbiota. Our results confirm and extend previous results from mouse experiments^10^,^11^ and indicate that macrolide use may have undesired effects on the developing microbiota of children, which may compromise the development of a healthy immune system and metabolism.

## Methods

### Study cohort

Our cohort consists of 236 Finnish children aged 2–7 years (median age 5 years), attending the same day-care centres at the time of the study. Register-based information and full background information was available for all children; 142 children donated fecal samples. The children are part of a larger cohort originally recruited for a probiotic trial^46^. Details on the recruitment and exclusion criteria are available in the original publication presenting the cohort^46^. Written informed consent was obtained from the parents and the local ethical committees approved the study. The children attended a health check in the beginning of the study, during which weight and height were measured. Based on the weight and height, BMI *z*-scores were calculated according to the LMS parameters obtained from the Centers of Disease Control and Prevention. Data on allergies were obtained from questionnaires filled out by the parents. Nearly all (>95%) of the children had received breastfeeding (>70% for at least 6 months), as this is highly promoted in Finland where mothers obtain leave of absence for up to 3 years with job guarantees. All children that developed asthma had received breastfeeding, and all but one received it for 6 months or more. The children received three daily meals at the day-care centre; the majority of their diet was thus largely similar. Furthermore, income inequality in Finland is low compared with other western countries^47^, suggesting that socio-economic differences were fairly small. A total of 257 fecal samples were obtained from 142 children who donated either a single sample (27 children) or two samples (115 children; samples were taken 7 months apart; Supplementary Fig. 1). This sub-cohort was utilized to analyse the association between antibiotic use and the intestinal microbiota.

### Antibiotic purchase and chronic illness data

In Finland, antibiotics are only available by prescription. Information on antibiotic purchases was obtained from the records of the Finnish Social Insurance Institution, which subsidizes health-care costs. We collected data on all antibiotics purchased for the study children from the date of birth to the date of donation of the last fecal sample. Individuals with chronic illnesses are eligible for special reimbursement of their drug purchases, and the eligibility information is stored in the national database. We collected data on diagnosed asthma and allergic dermatitis (until the end of 2012), the most common developing chronic illnesses in the study cohort. The antibiotics were grouped into penicillins (amoxicillin with or without clavulanic acid and penicillin V), macrolides (azithromycin and clarithromycin), first-generation cephalosporins and sulphonamide–trimethoprim. The latter two groups of antibiotics were very seldom used, and therefore we focused on the penicillin and macrolide use. Azithromycin and clarithromycin became available in Finnish ambulatory care in 1994 and 1996, respectively, that is, before the birth of the study children. In our cohort, 50% of all antibiotic purchases were penicillins, 24% macrolides and the rest cephalosporins and sulfonamide–trimethoprim. These figures correspond to the population level data from Finland^48^ and other countries as well^49^.

Based on the parent-reported symptoms during the 7-month interval, we were able to assign potential indications for the use of antibiotics during the interval period. Respiratory infections were the most common apparent indication, occurring in association with 88% of the courses; other infections (mainly of the urinary tract) were associated with 5% and no concurrent reported symptoms with 7% of the courses. The antibiotic courses were not prescribed for gastrointestinal infections; pediatric gastrointestinal infections in Finland are nearly always viral and hence not treated with antibiotics. According to the physician in charge of the study, concurrent symptoms, indications for the antibiotics, symptom severity or disease history of the children receiving macrolides during the interval period did not differ from those receiving penicillins. Macrolides were typically prescribed due to presumed penicillin allergy or parents' request owing to easy administration or previous experience.

Associations between health and antibiotic use were analysed using the full cohort of 236 children. Associations between antibiotic use and BMI *z*-score were assessed using Pearson correlations. Associations between antibiotic use during the first 2 years of life and asthma (*N*=15), and allergic dermatitis (*N*=5) were assessed using the Fisher's test.

### Processing of the fecal samples

The fecal samples were collected at home and transported immediately to the study centre for storage in −70 °C. DNA was extracted from the fecal samples using the Promega Wizard Genomic DNA Purification Kit as described^50^. Concentration of DNA was measured with NanoDrop and adjusted to 10 ng ml^−1^.

### Sequencing

Bacterial composition was investigated using 454 Titanium sequencing of the V4–V6 region of the 16S rRNA gene (primers S-D-Bact-0564-a-S-15/S and Univ-1100-a-A-15 that have been recommended for pyrosequencing^51^). The sequences were filtered for chimeras with the Uchime programme^52^. Reads shorter than 501 bp were filtered out. We discarded four samples with <1,000 reads. Read numbers were not equalized by rarefying as this procedure causes a significant and unnecessary loss of data^53^. After pre-processing, we had a total of 2,262,107 reads from 257 samples (on average 8,801 reads per sample, range 1,469–14,653 reads per sample). *De novo* operational taxonomic unit (OTU) picking was done using Qiime version 1.8.0 (ref. 54). The Uclust method was used to map the OTUs to the Greengenes 13.8 taxonomy. To avoid a batch effect, we normalized the data following a method we have developed earlier^55^. The number of reads was controlled for by keeping it as a covariate in all relevant models. Number of reads was not associated with observed diversity or richness (Supplementary Fig. 5).

Shotgun metagenomic sequencing with the Illumina HiSeq2000 platform was conducted on 14 samples derived from children, which were all treated with macrolide antibiotics during the 7-month interval period. Analysis of the antibiotic resistome was conducted as detailed previously^56^. In brief, by using the MOCAT pipeline, Illumina sequencing reads, following trimming and quality control, were mapped to a catalogue of 3.3 M gene sequences, previously assembled from publically available human gut microbial metagenomes. Out of these reference genes, we identified members of the antibiotic-resistant gene families annotated in the ARDB database. This was done by first augmenting database with further homologues from an in-house collection of 3,496 bacterial genomes from GenBank, and then identifying homologues of the resulting gene set among the reference genes using the ARDB family-specific sequence identity thresholds for family assignment, using NCBI BLAST. As a result, the abundance of sequence material coming from reads mapping to members of each antibiotic-resistant gene family could be quantified. Reference gene sequences were similarly mapped to functional modules from the KEGG database to allow testing for changes in the relative abundance of genes encoding different metabolic pathways.

### Culture-based antibiotic sensitivity testing

Phenotypic antibiotic-resistant analysis was conducted by cultivating bacteria anaerobically from 80 fecal samples as mixed cultures. Bacteria were grown as dilution series on Brain-Heart Infusion agar (Oxoid) with or without 10 mg ml^−1^ erythromycin (a macrolide antibiotic). Colonies were counted on each plate after 2-day incubation at 37 °C, and the fraction of resistant colonies was calculated as the number of colony-forming units (c.f.u.'s) per 1 g of sample on the antibiotic plate divided by the c.f.u. on the control plate.

### Real-time PCR analysis

The metagenomic analysis indicated that apart from an increase in macrolide resistance, macrolide use was associated with a decrease in bile-salt hydrolase genes. We therefore conducted qPCR analyses to quantify the abundances of these genes in a total of 130 DNA samples from 117 children. Quantification of bacterial genes *bsh, ermB* and *ermF* were performed with primers specified in Supplementary Table 4. The primers for *ermB* and *ermF* genes have been previously published^57^. We designed the *bsh* primers to target this gene in the dominant phyla Firmicutes and Bacteroidetes. We focused on species that have confirmed bile-salt hydrolase activity and no penicillin V amidase activity^32^ to avoid co-amplification of these highly homologous genes. Hence, we designed primers for amplification of *bsh* genes encoded by *Bacteroides ovatus, Ruminococcus obeum* and *Eubacterium ventriosum* based on sequence data obtained from GenBank using BLAST. The optimal primer concentrations and annealing temperatures were determined for each assay, and the specificity of the primer pairs was verified by melting curve analysis as well as agarose gel electrophoresis to confirm the correct product size. Reactions were run in triplicate on iCycler iQ Real-Time PCR Detection System (Bio-Rad, USA) in a volume of 25 μl. Each reaction contained 1 × HOT FIREPol EvaGreen qPCR Mix Plus (Solis Biodyne, Estonia.), 0.5 μM of each primer and 25 ng of fecal DNA. The amplification protocol involved one cycle at 95 °C for 5 min for initial denaturation followed by 40 cycles of denaturation at 95 °C for 20 s, primer annealing for 20 s, extension at 72 °C for 30 s and an additional incubation step at 80–82 °C for 30 s to collect the fluorescence data. The mean Ct value per sample (after excluding replicates with Ct values that differed >0.5) was used as a proxy for relative abundance of the target gene.

### Antibiotic use study groups

The fecal samples were grouped based on antibiotic use (Supplementary Table 1). The control group (group C) included samples from children that had not used antibiotics for a minimum of 2 years, and whose total lifetime antibiotic use was <1 course per year. The children who had not used antibiotics for at least 2 years but had a high total lifetime use (at least 1 course per year of life on average), that is, had used antibiotics frequently during early life but not recently, were assigned to the early-life group (E). The group M6 consisted of samples from children that had used macrolide antibiotics within 6 months before sample donation. The group M12 consisted of samples from children that had used macrolide antibiotics within 6–12 months before sample donation. The group M24 consisted of samples from children that had used macrolide antibiotics within 12–24 months before sample donation. The group P6 consisted of samples from children that had used penicillin antibiotics within 6 months before sample donation. The group P12 consisted of samples from children that had used penicillin antibiotics within 6–12 months before sample donation. The group P24 consisted of samples from children that had used penicillin antibiotics within 12–24 months before sample donation.

### Indices calculated based on the microbiota

Microbiota richness was calculated as the number of species-level OTUs (97% similarity cutoff) detected. Microbiota diversity was calculated as the inverse Simpson diversity index based on the species-level data. PCoAs were conducted using the genus-level data and Bray–Curtis dissimilarities. The associations of the first principal components with background variables such as antibiotic use were visualized by inverse distance-weighted interpolation of the variable values over the component space. Microbiota maturity index, which has previously been shown to differentiate between healthy children and those with health issues^58^,^59^, was calculated as the first principal coordinate from a PCoA using only significantly age-associated genus-level taxa in the control and early-life groups (Supplementary Table 2). The group differences in microbial richness (counts of taxa) were evaluated using negative binomial models and the differences in microbiota maturity (normally distributed) using analysis of variance.

### Association between antibiotic use and bacterial genera

Generalized linear models with negative binomial distribution (function glm.nb in the library MASS in R) were used to identify individual bacterial taxa that were associated with antibiotic use, either as being significantly different in the antibiotic user groups compared with the control group or significantly linearly associated with the total lifetime number of macrolide or penicillin courses (per years of life). We focused only on the 48 genera that we detected in >30% of the samples, in order to avoid unnecessary *P* value testing and unreliable results. The detected abundance of rare genera is likely to be influenced considerably by chance and therefore less reliable than the abundance of common genera. The main focus of interest was to assess how the different groups (Supplementary Table 1) differ in their microbiota composition. However, to control for potentially confounding factors, we included in all models the following covariates: number of sequencing reads, age of the child, BMI *z*-score of the child (which is likely to reflect lifestyle and diet at home), health status (parent-reported or diagnosed allergies, diagnosed asthma or no known diseases) and lifetime antibiotic use (when comparing antibiotic user groups) or antibiotic user group (when assessing the cumulative effect of lifetime antibiotic use). False disovery rate (FDR)-corrected *P* values^60^ were used to correct for multiple testing. Bacterial taxa, whose abundance was significantly different (FDR-corrected *P* value <0.15) from the control group after controlling for all the above-mentioned variables, were considered potentially influenced by antibiotic use.

### Microbiota–health association analysis

To determine whether the asthmatic cases had distinct features in their microbiota composition, we selected the samples from all cases with current or developing asthma (*N*=8), and two age- and antibiotic use history-matched healthy controls for each (*N*=16). For analysis of the microbiota–overweight association, we selected the samples from all cases with BMI *z*-score >2 (*N*=9) and two (if available) age and antibiotic use history-matched normal-weight controls for each (*N*=16). For two overweight individuals, only one matching control case was available. For the asthma association analysis, we calculated the average microbiota composition between the first and second time point samples, if two samples were available. For the overweight analysis, we used only the first time point sample, as the weight and height were only recorded once at the beginning of the study. To find the bacterial groups that best distinguished asthma or overweight cases from healthy or normal-weight cases, we first fitted binomial models with asthma or overweight as the response variable, and each genus-level bacterial group separately as an explanatory variable. We used *P* values from these models as indicators of discriminating potential, and selected all bacterial groups with *P*<0.2 for further analysis. These groups were combined as explanatory variables in one model. AIC-based stepwise model selection was then used to find the set of bacterial groups that best discriminated between the asthma/overweight and healthy/normal-weight cases.

All statistical analyses were conducted with the programme R (ref. 61) by using the packages vegan^62^ for the ordinations and diversity analyses, and MASS^63^ for negative binomial models.

## Additional information

**Accession codes:** The reported metagenome data and 16S rDNA amplicon sequences have been deposited in the European Nucleotide Archive (ENA) under the study name PRJEB11685.

**How to cite this article:** Korpela, K. *et al*. Intestinal microbiome is related to lifetime antibiotic use in Finnish pre-school children. *Nat. Commun.* 7:10410 doi: 10.1038/ncomms10410 (2016).

## Supplementary Material

Supplementary Information Supplementary Figures 1-5, Supplementary Tables 1-4 and Supplementary References
