# Supplementary material for: Intestinal microbiome is related to lifetime antibiotic use in Finnish pre-school children
Source: Nat Commun. 2016 Jan 26;7:10410. doi: 10.1038/ncomms10410 (PMC4737757; doi:10.1038/ncomms10410)
Supplement: Supplementary Information — Supplementary Figures 1-5, Supplementary Tables 1-4 and Supplementary References [file ncomms10410-s1.pdf]

Supplementary Information

Supplementary Figures

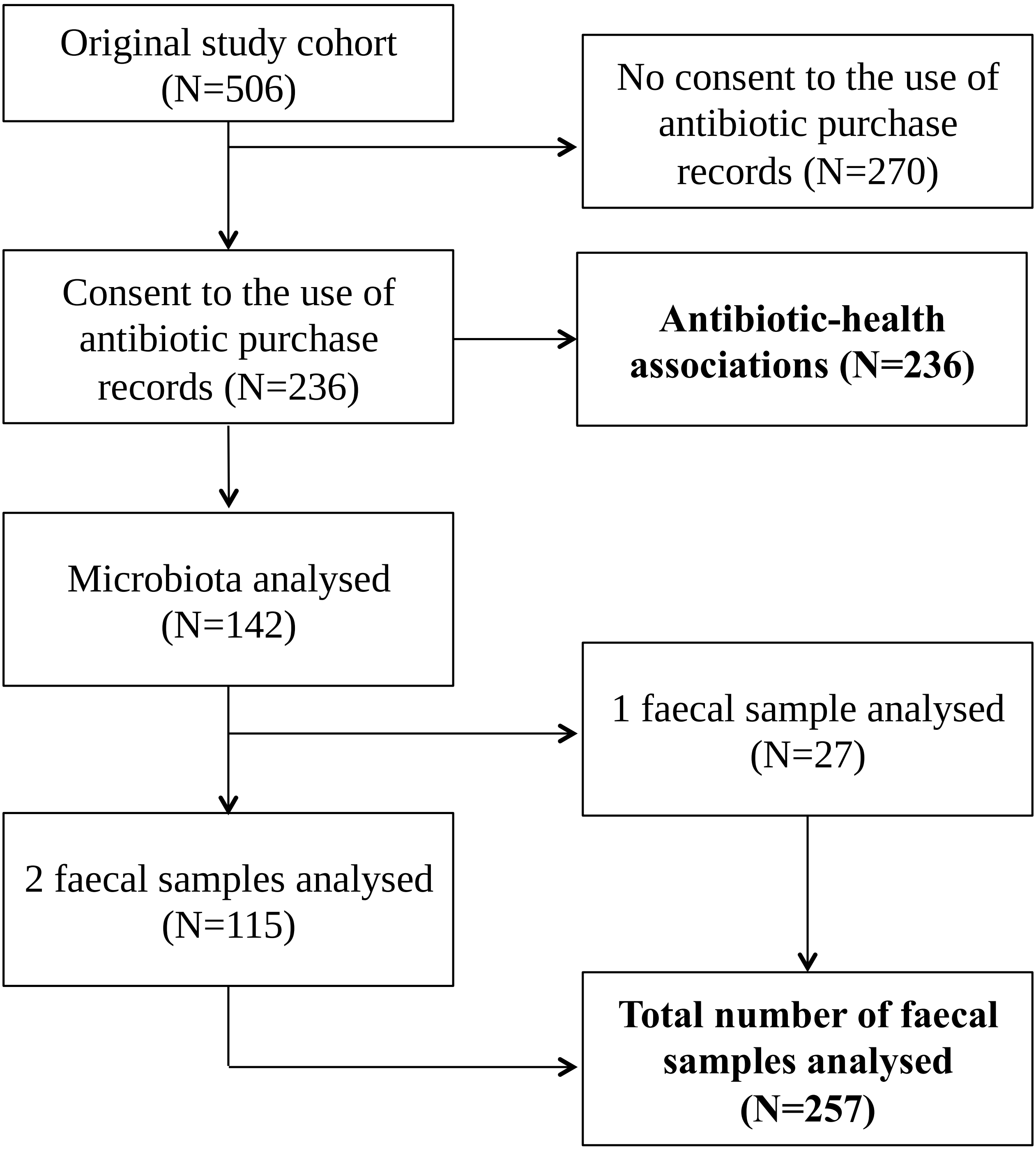

Supplementary Figure 1. Sample flow chart.

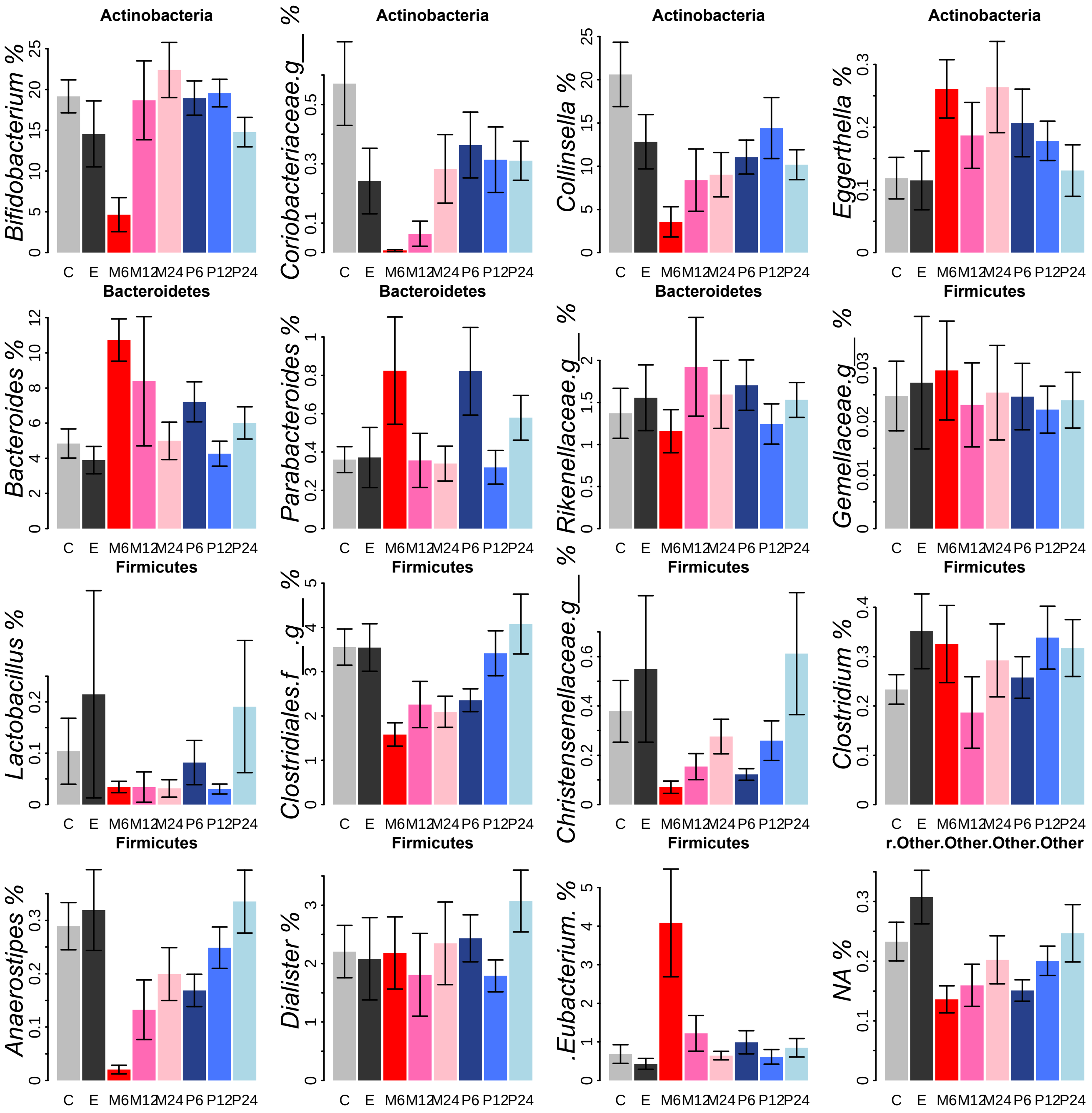

Supplementary Figure 2.

Significant genus-level microbiota differences between groups. Average abundance (± standard error) of genus-level groups differing significantly between the control group and at least one of the antibiotic-exposed groups (see Supplementary Table 3). The phylum is indicated on top of the panel.

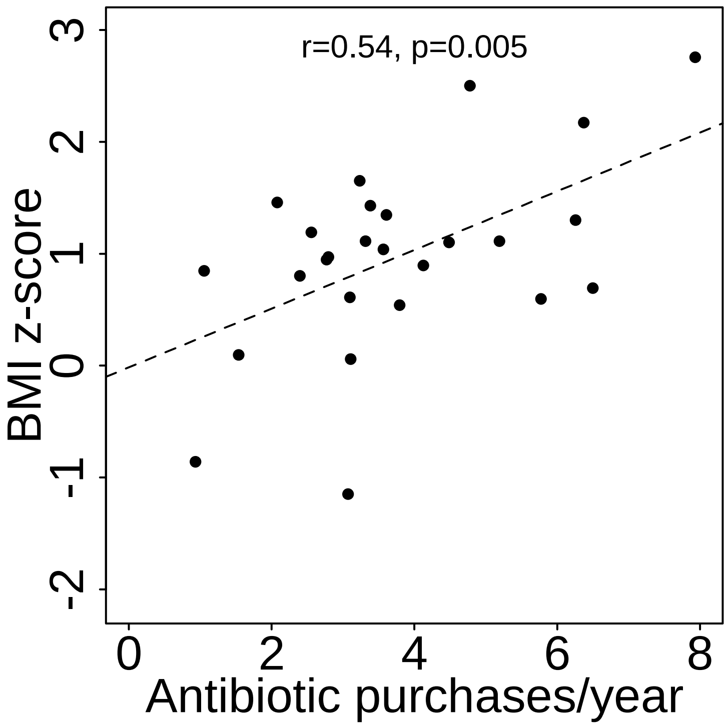

Supplementary Figure 3. BMI and average lifetime antibiotic use. Associations between average lifetime antibiotic use (average number of antibiotic purchases per year of life from birth to BMI measurement day) and BMI z-score in 2-6 year old children who had received more than 2 courses of macrolides by the age of 2 years. One purchase/year would indicate that a 6 year old child had a total of 6 courses in their lifetime while 8 purchases/year would indicate that a 6 year old child had a total of 6*8=48 courses in their lifetime. For a 2-year old, the same total lifetime values would be 2 courses and 2*8=16 courses, respectively.

A

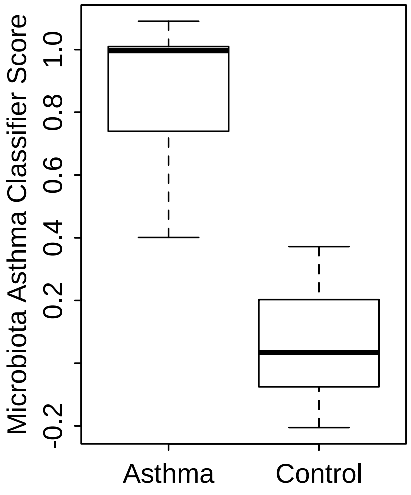

	Control	Asthma
Blautia	6.9 (15/16)	10.7 (8/8)
Rothia	0.01 (8/16)	ND (0/8)
Coprobacillus	0.17 (15/16)	0.53 (7/8)

B

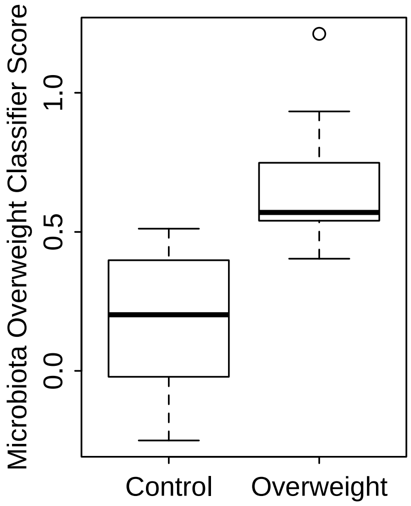

	Control	Overweight
Clostridium (Erysipelotrichacaea)	0.05 (6/16)	ND (0/9)
Clostridium (Clostridiacaea)	0.44 (13/16)	0.18 (6/9)
Enterococcus	0.02 (7/16)	0.03 (2/9)
Akkermansia	3.66 (12/16)	1.14 (7/9)

Supplementary Figure 4. Microbiota-based classifiers for asthma (panel A) and overweight (panel B). The table entries show the mean abundance of the classifying bacterial groups (% of total microbiota) in those children where the bacterial group was present, and the prevalence of each group in the controls and asthmatic or overweight children (in brackets). ND indicates below detection level (< 0.001 %). Asthma controls were healthy age- and antibiotic use-matched children. Overweight controls were normal weight age- and antibiotic use –matched children. In both cases, the p-value of the classifier score difference between groups is <0.0001 (binomial generalized linear model).

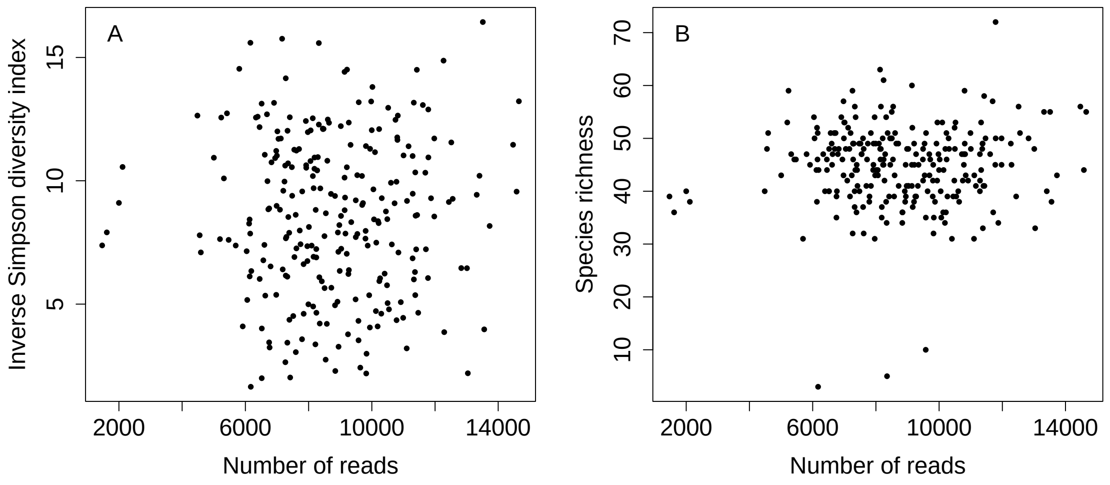

Supplementary Figure 5.
Effect of the number of reads. Microbiota diversity (A) and richness (B) compared to the number of reads obtained for each sample after quality control and filtering. These figures indicate that the observed diversity and richness were not correlated with the sequencing depth, i.e. that more reads did not result in more observed taxa within the range of reads used in this study. For this reason, we included all reads from all samples instead of rarefying the number of reads.

Supplementary Tables

Supplementary Table 1.
Characteristics of the groups used in the microbiota analysis.

	C	E	M6	M12	M24	P6	P12	P24
N	43	14	27	13	21	53	47	43
Cases with asthma	0	0	4	2	2	2	3	1
Cases with allergies	7	1	4	2	6	13	11	11
Mean BMI	16.5 (1.9)	17.0 (1.4)	16.7 (1.7)	16.7 (1.5)	16.7 (1.3)	16.6 (1.4)	16.7 (1.5)	16.9 (2.3)
Mean duration of breastfeeding (months)	9.0 (4.8)	9.0 (7.0)	9.9 (6.4)	5.9 (4.1)	8.4 (6.6)	9.0 (4.9)	8.1 (5.6)	7.8 (4.8)
Mean age (years)	5.7 (1.3)	5.5 (0.9)	4.5 (1.5)	4.1 (1.3)	4.8 (1.3)	4.2 (1.4)	4.8 (1.4)	4.8 (1.4)
Mean number of lifetime antibiotic courses	3.2 (2.1)	10.0 (4.3)	12.6 (6.6)	8.3 (5.7)	9.3 (5.6)	9.2 (6.8)	7.5 (5.2)	6.2 (4.8)
Mean number of months since the last antibiotic course	37.0 (11.3)	28.4 (9.9)	3.1 (1.9)	8.5 (1.8)	15.4 (4.5)	3.3 (1.9)	8.9 (2.1)	16.9 (4.6)

C = control group, no antibiotics for 2 years and in total <1 course/year. E = early-life exposure group; no antibiotics for 2 years and >1 course/year in total. M6 = macrolide course within 6 months; M12 = macrolide course within 6-12 months; M24 = macrolide course within 12-24 months. P6, P12, P24 = penicillin courses within 6, 6-12, and 12-24 months, respectively. Standard deviations are given in brackets.

Supplementary Table 2.
Age-associated genus-level bacterial taxa in the control and early-life groups.

Genus-like group	FC	p
Actinobacteria/*Actinobacteria/Actinomycetales/Actinomycetaceae/Actinomyces*	0.81	2.07E-21
Actinobacteria/*Actinobacteria/Actinomycetales/Micrococcaceae/Rothia*	0.80	5.55E-04
Actinobacteria/*Coriobacteriia/Coriobacteriales/Coriobacteriaceae/Adlercreutzia*	1.39	0.00E+00
Actinobacteria/*Coriobacteriia/Coriobacteriales/Coriobacteriaceae/Atopobium*	0.62	2.68E-09
Actinobacteria/*Coriobacteriia/Coriobacteriales/Coriobacteriaceae/Collinsella*	1.19	0.00E+00
Bacteroidetes/*Bacteroidia/Bacteroidales/Porphyromonadaceae/Parabacteroides*	0.67	0.00E+00
Bacteroidetes/*Bacteroidia/Bacteroidales/Prevotellaceae/Prevotella*	0.66	5.75E-287
Bacteroidetes/*Bacteroidia/Bacteroidales/S24.7/*	0.71	2.81E-227
Bacteroidetes/*Bacteroidia/Bacteroidales/.Barnesiellaceae./*	1.33	7.33E-41
Bacteroidetes/*Bacteroidia/Bacteroidales/.Odoribacteraceae./Odoribacter*	0.91	6.68E-10
Cyanobacteria/4C0d.2/YS2//	0.70	9.35E-16
Firmicutes/*Bacilli/Gemellales/Gemellaceae/*	1.08	3.82E-04
Firmicutes/*Bacilli/Lactobacillales/Leuconostocaceae/Leuconostoc*	0.73	3.98E-39
Firmicutes/*Bacilli/Turicibacterales/Turicibacteraceae/Turicibacter*	0.77	1.74E-18
Firmicutes/*Clostridia/Clostridiales/Dehalobacteriaceae/Dehalobacterium*	0.85	1.35E-09
Firmicutes/*Clostridia/Clostridiales/Peptococcaceae/rc4.4*	0.70	6.35E-29
Firmicutes/*Clostridia/Clostridiales/Ruminococcaceae.Other*	1.24	2.61E-18
Firmicutes/*Clostridia/Clostridiales/Veillonellaceae/Dialister*	0.87	0.00E+00
Firmicutes/*Clostridia/Clostridiales/Veillonellaceae/Phascolarctobacterium*	0.91	1.11E-09
Firmicutes/*Clostridia/Clostridiales/.Tissierellaceae./Peptoniphilus*	1.51	4.00E-06
Firmicutes/*Erysipelotrichi/Erysipelotrichales/Erysipelotrichaceae/Bulleidia*	0.72	4.01E-17
Proteobacteria/*Alphaproteobacteria*/RF32//	0.73	1.77E-37
Proteobacteria/*Deltaproteobacteria/Desulfovibrionales/Desulfovibrionaceae/Bilophila*	1.18	2.27E-92
Tenericutes/*Mollicutes*/RF39//	0.65	1.06E-251

Estimates based on negative binomial models. FC = Fold change per year of age. The FC values indicate the change associated with each accumulating year of age. For example, *Actinomyces* is estimate to decline by 20% each year during the preschool age (2-7 years). Conversely, *Adlercreutzia* is estimated to increase each year by 39%.

Supplementary Table 3.

Fold changes of bacterial taxa at three different levels, compared to the control group.

Phylum	Order	Genus-like group	E	M6	M12	M24	P6	P12	P24
Unassigned				0.55			0.65		
Actinobacteria				0.31					
	Bifidobacteriales			0.23					
		Bifidobacterium		0.23					
	Coriobacteriales			0.26					
		Unassigned *Coriobacteriaceae*		0.01					
		Collinsella	0.61	0.28	0.71	0.54	0.82	0.88	0.60
		Eggerthella		11.44					
Bacteroidetes				1.74			1.46		
	Bacteroidales			1.74					
		Bacteroides		2.04					
		Parabacteroides	1.15	1.58	1.05		2.54	0.96	1.61
		Unassigned *Rikenellaceae*	1.27	0.82	1.29	1.106	1.15	0.92	1.13
Firmicutes									
	Gemellales		1.3		0.79		0.84	0.83	0.83
		Unassigned *Gemellaceae*			0.79		0.84	0.82	0.84
	Lactobacillales		2.5	2.29	2.28	2.00			
		Lactobacillus		0.12		0.05	0.11	0.09	
	Erysipelotrichales			1.91					
		Eubacterium		3.52					
	Clostridiales								
		Unassigned		0.48					
		Unassigned *Christensenellaceae*		0.22					
		Clostridium		2.68					
		Anaerostipes		0.07					
		Dialister	0.91	0.97	0.85	1.07	1.1	0.81	1.43
Proteobacteria				1.96					

E = early-life exposure group; no antibiotics for 2 years and >1 course/year in total. M6 = macrolide course within 6 months; M12 = macrolide course within 6-12 months; M24 = macrolide course within 12-24 months. P6, P12, P24 = penicillin courses within 6, 6-12, and 12-24 months, respectively. The cases where the FDR-corrected p-value is >0.15, the fold change is set to 1. Where the fold change deviates from 1, the FRD-corrected p-value is <0.15 (the raw p-value is <0.004 for the genus-level, <0.008 for the orders, and <0.02 for the phyla). All values that are shown are significant after correcting for multiple p-value testing.

Supplementary Table 4.
Primers for bile salt hydrolase and macrolide resistance genes used in quantitative PCR analysis.

Primer name	Sequence
ObeumBSH_F	5′-CGCCATGTTGGCGATATGAA-3′
ObeumBSH_R	5′-TGCTAATGGCAATTGCTCGC-3′
OvatusBSH_F	5′-GGCATCCAACGTATGGGACA-3′
OvatusBSH_R	5′-AAGGTAGACTCCGGTCCTCC-3′
VentriosumBSH_F	5′-CCGGTTTGCCGGGAAGATAA-3′
VentriosumBSH_R	5′-CCACCTGGCTGACTTCTGTT-3′
ermB_F*	5′-GAT ACC GTT TAC GAA ATT GG-3′
ermB_R*	5′-GAA TCG AGA CTT GAG TGT GC-3′
ermF_F*	5′-CGA CAC AGC TTT GGT TGA AC-3′
ermF_R*	5′-GGA CCT ACC TCA TAG ACA AG-3′

Primers marked with * are from Chen *et al.*[1].
All other primers were designed specifically for this study.

Supplementary References

1. Chen, J., Yu, Z., Michel, F. C., Jr, Wittum, T. & Morrison, M. Development and application of real-time PCR assays for quantification of erm genes conferring resistance to macrolides-lincosamides-streptogramin B in livestock manure and manure management systems. *Appl. Environ. Microbiol.* **73**, 4407-4416 (2007).
